# Promotion of Melanoma Progression through MCM4-Induced Immune Suppression and Polarization of Macrophages by Carcinogenic Exosomes

**DOI:** 10.2174/0115680096357750250507073948

**Published:** 2025-05-14

**Authors:** Xuewei Zhang, Sirong Liu, Deni Kang, Ronghua Yang

**Affiliations:** 1 Department of Burn and Plastic Surgery, Guangzhou First People's Hospital, Guangzhou Medical University, Guangzhou, 510000, Guangdong, China

**Keywords:** Malignant melanoma, minichromosome maintenance 4, macrophages, exosomes, tumor microenvironment

## Abstract

**Objective:**

This study aims to provide a comprehensive understanding of the crucial role of MCM4 in melanoma progression regarding the regulatory communication between macrophages and cancer cells mediated by extracellular vesicles.

**Methods:**

Initially, a preliminary analysis was conducted using the Tumor Immune Estimation Resource (TIMER) database. Subsequently, the role of MCM4 knockdown on the polarization of THP-1 and RAW264.7 macrophages was observed. Finally, the biological functionalities of exosomes derived from A375 cells overexpressing MCM4 on normal melanocytes (HEM-L) were explored.

**Results:**

On the one hand, MCM4 knockdown resulted in the upregulation of M1 macrophage markers and downregulation of M2 macrophage markers, indicating that MCM4 could facilitate polarization of macrophages toward the M2 phenotype and suggesting its oncogenic potential. On the other hand, MCM4 overexpression in melanocytes increased the secretion of exosomes, enhancing the proliferation, clonogenic, and DNA synthesis abilities of normal melanocytes. In addition, MCM4 overexpression-induced secretion of exosomes promoted the migration and invasion capabilities of normal melanocytes.

**Conclusion:**

Exosomes secreted by MCM4-overexpressed melanocytes could stimulate their proliferation, migration, and invasion abilities. MCM4 promoted M2 polarization of macrophages, indicating its crucial role in tumor microenvironment formation and thereby facilitating tumor development.

## 1 INTRODUCTION

The tumor microenvironment (TME) of malignant solid tumors mainly consists of tumor-associated immune cells, tumor-associated fibroblasts, endothelial cells, and extracellular components such as growth factors, hormones, cytokines, and the extracellular matrix [1-4]. Typically, the TME plays a very crucial role in maintaining the growth, invasiveness, and malignant progression of tumor cells, as well as their responsiveness to various chemotherapeutics [2, 5-7]. Among various cell types, the tumor-associated macrophages (TAMs) [8], recruited from circulating monocytes and infiltrating tumor tissues, are the most abundant population of tumor-infiltrating immune cells in the TME [3, 9, 10]. Along this line, the macrophages with high plasticity can be classified into two main phenotypes: the classically activated M1 subtype and the alternatively activated M2 subtype [11, 12]. M1 macrophages significantly exert anti-tumor effects through the production of effector molecules and pro-inflammatory cytokines, such as interleukin (IL)-1, IL-6, and interferon-gamma (IFN-γ) [13]. Contrarily, M2-polarized macrophages promote tumor activity by producing anti-inflammatory and immune-suppressive cytokines (for instance, IL-2), as well as transforming growth factor-beta (TGF-β). M2-polarized TAMs often facilitate tumor growth, angiogenesis, and metastasis, which is associated with poor clinical outcomes in many human cancers [14-16]. Patients with high infiltration of M2-polarized TAMs often have worse overall survival (OS) and progression-free survival (PFS) rates compared to those with low infiltration of M1-polarized TAMs [9, 13, 14, 17]. Therefore, M2-polarized TAMs might serve as potential biomarkers and promising targets for tumor immunotherapy.

To this end, exosomes, small vesicles derived from cells that are enclosed in a lipid bilayer, carry various bioactive molecules, including proteins, polysaccharides, lipids, deoxyribonucleic acid (DNA), ribonucleic acid (RNA), and metabolites, and mediating intercellular communication as well as material exchange characteristics [18, 19]. These exosomes significantly contribute to the information and material exchange between various tumor cells and other stromal cells or immune cells in the TME [20-22]. Previous reports demonstrated that tumor-derived exosomes could influence the behavior of surrounding normal cells, including altering their proliferation, migration, differentiation, and apoptosis. In addition, these vesicles could impact the responses of the immune system, assisting tumor immune evasion. Furthermore, tumor-derived exosomes can regulate distant tissues, creating a favorable environment for tumor cell metastasis and conferring resistance to chemotherapy [23-25].

Typically, the Minichromosome Maintenance (MCM) protein family is composed of highly conserved hexameric complexes of DNA-binding proteins, including six subtypes, such as MCM2, MCM3, MCM4, MCM5, MCM6, and MCM7 [26]. These proteins assemble as a hexameric complex around DNA, in which any dysfunction directly affects the DNA replication system, contributing to the development of various metabolic (cancer) and autoimmune diseases. Previous studies demonstrated that MCM6 might promote carcinogenesis through its impact on immune escape based on anaplastic oligodendroglioma (AO) and systemic lupus erythematosus (SLE). These findings could suggest a close relationship between MCM6 and immune responses [27, 28]. Nonetheless, the immune expression pattern of MCM4 in melanoma and its impact on the TME has not been reported yet.

Motivated by these considerations, this study aims to provide a comprehensive understanding of the crucial role of MCM4 in melanoma progression regarding the regulatory communication between macrophages and cancer cells mediated by extracellular vesicles. Initially, preliminary analysis was conducted using the Tumor Immune Estimation Resource (TIMER) database. Subsequently, the role of MCM4 knockdown on the polarization of THP-1 and RAW264.7 macrophages was observed. Finally, the biological functionalities of exosomes derived from A375 cells overexpressing MCM4 on normal melanocytes (HEM-L) were explored.

## 2 EXPERIMENTAL/ METHOD SECTION

### 2.1 Database Analysis

The TIMER database (https://cistrome.shinyapps.io/timer/) analyzes the immune cell infiltration in various cancers through bioinformatics analysis. This database employs statistically validated methods based on pathological examination. It significantly estimates the immune cell infiltration in cancer samples, including neutrophils, macrophages, dendritic cells, B cells, and CD4^+^/CD8^+^ T cells. Further, the correlation can be explored between the expression levels of specific genes and the immune system's infiltration in the TME. Considering these abilities, the differential expression levels of MCM4 in melanoma were initially evaluated using the TIMER database. Subsequently, the relationship between MCM4 expression and the infiltration levels of specific immune cell subgroups was explored. Finally, the correlation between the expression levels of specific immune cell subgroup markers was determined.

### 2.2 Cell Culture and Transfection

HEM-L, RAW264.7, THP-1, and A375 cells purchased from American Type Culture Collection (ATCC, Manassas, USA) were cultured in Dulbecco's modified eagle medium (DMEM, ThermoFisher, Waltham, USA) supplemented with 10% fetal bovine serum (FBS, Sangon Biotech, Inc., Shanghai, China) and 1% penicillin-streptomycin (PS) and incubated at 37°C with 5% CO_2_. Further, the transfection of RAW264.7, THP-1, or A375 cells was performed by incubating with MCM4-small interfering RNA (siRNA) or MCM4 overexpression pcDNA3.1 plasmid (Gemma Gene Co., Ltd., Suzhou, China) for definite time intervals. The siRNA target sequences applied in this study are presented in supplementary material (Table **SI**).

### 2.3 Extraction of Extracellular Vesicles

The extraction of extracellular vesicles was performed using the following procedure. Initially, the collected cell culture supernatant was transferred to a centrifuge tube and centrifuged at 3000 g for 5 min at 4°C. After discarding the pellet, the supernatant was transferred to a new centrifuge tube for further purification of extracellular vesicles. Then, the cell-free supernatant was transferred to high-speed centrifugation, typically at around 12,000 g, for 30 min to 1 h. The pellet containing extracellular vesicles was washed 1-2 times using a suitable washing phosphate-buffered saline (PBS) buffer. Finally, the pellet with extracellular vesicles was dissolved in an appropriate buffer for subsequent analysis.

### 2.4 Characterization of Extracellular Vesicles

The morphology of the formed extracellular vesicles was observed using transmission electron microscopy (TEM). The fixed extracellular vesicles were prepared by adding an appropriate amount of sample onto copper grids and allowing them to settle at room temperature for 10 min. Further, a 2% phosphotungstic acid solution adjusted to pH = 6.5 was added to the copper grids for staining the extracellular vesicles for 2 min at room temperature. After air-drying, the samples were observed using an electron microscope at a voltage of 120 kV. In addition, Western blot analysis was performed to detect specific protein markers in extracellular vesicles. The vesicle size and particle abundance were determined as a part of nanoparticle tracking analysis using the NanoSight LM10 system (Malvern Instruments, Malvern, UK).

### 2.5 Western Blot Analysis

Initially, cells were homogenized in the cold radio-immunoprecipitation assay (RIPA) buffer (Beyotime Biotechnology Inc., Shanghai, China) to extract protein. After centrifugation at 13000 rpm for 20 min, the extracted protein was quantified using the bicinchoninic acid (BCA) protein assay kit (Bio-Rad, Hercules, USA). Further, 50 μg of the extracted protein was separated with sodium dodecyl-sulfate polyacrylamide gel electrophoresis (SDS-PAGE) and then transferred onto a polyvinylidene difluoride (PVDF) membrane. After blocking the membranes at room temperature for 1 h using the skimmed milk, these membranes were incubated with various primary antibodies, including CD81, CD63, PCNA, C-MYC, and MMP2 (Abcam Co. Ltd., Cambridge, UK), overnight at 4°C. Then, the membrane was incubated with horse-radish peroxidase (HRP)-conjugated goat anti-rabbit or anti-mouse immunoglobulin G (IgG) antibodies for 1 h at room temperature. Finally, an enhanced chemiluminescence (ECL) detection kit (Tsea Biotech Co., Shanghai, China) was employed to visualize the protein bands. Additional information regarding the BCA experiment is included in Supplementary Information. The details and dilution of the utilized antibodies including primary and secondary are provided in the supplementary information (Table **SII**).

### 2.6 Cell Counting Assay

To determine the proliferation abilities of the cells, a CCK-8 assay was performed as previously described [18]. Briefly, cells at a density of 4000 cells/well were seeded in a 96-well plate and incubated overnight for proper cell attachment. After inoculation of the corresponding plasmids, the cells were further cultured in an incubator for 0 h, 24 h, 48 h, and 72 h. Subsequently, each well was supplemented with 10 µL of CCK-8 working solution (Abcam Co. Ltd.). Subsequently, the cells were incubated additionally for a period of 4 h. Eventually, the proliferation rate of the cells was determined by scanning the plates at an absorbance of 450 nm (OD) using a microplate reader.

### 2.7 Clone Forming Assay

Briefly, cells at a density of 800 cells/well were seeded and cultured in a 12-well plate. After a two-week incubation period, cells were subjected to PBS wash, followed by a 15-minute fixation using 10% formaldehyde at room temperature. Subsequently, the cells were stained using crystal violet (Hunan Yunbang Biotechnology Co., Ltd., Changsha, China) for 15 min. The formed cell colonies were finally enumerated using an optical microscope (Olympus, Tokyo, Japan).

### 2.8 Cell Wound Scratch Assay

Cells at a density of 4 × 10^5^ cells/well were seeded in a 6-well plate. Further, the intersection points were marked with a 200 µL medium tip on the lid of a 6-well plate. Further, the cells were given washes for three rounds and added with serum-free DMEM to the wells. Finally, photographs were captured using an optical microscope after the corresponding incubation periods (0, 24, and 48 h) at the marked cross points, respectively.

### 2.9 Transwell Assay

To measure the migration ability, the Transwell assays were performed using Transwell^®^ cell culture plates (Corning Inc., Corning, USA). Briefly, the cell density was initially adjusted to 5 × 10^5^ cells/ml. Further, 200 μl of the cell suspension was supplemented in the Transwell chamber, and the cells were cultured conventionally for 12-48 h. Then, the well was fixed using methanol for 30 min. Subsequently, the fixed cells were stained with 0.1% crystal violet for 20 min. Finally, the cells were observed in five fields under a microscope (Olympus, Tokyo, Japan) at 200× magnification and counted.

### 2.10 Statistical Analysis

The data for statistical analysis were presented as mean ± standard deviation (S.D.). Statistical analyses were performed using the GraphPad Prism software (version 9). All cell-related experiments were independently replicated thrice and analyzed employing a two-way analysis of variance (ANOVA) and *t*-test to compare the differences among groups. It should be noted that the statistical significance of all tests performed was determined at a defined level of *p* < 0.05.

## 3 RESULTS

### 3.1 MCM4 Expression Regulates Immune Cell Infiltration in Melanoma

Initially, the TIMER database was employed to explore the correlation between MCM4 expression and infiltration of B cells, CD4^+^ T cells, CD8^+^ T cells, and macrophages in cutaneous melanoma and metastatic cutaneous melanoma (Fig. **1**). On the one hand, it was observed that the MCM4 expression in the cutaneous melanoma was significantly correlated with the levels of CD8^+^ T cells (partial.cor = 0.189, *p* = 6.87e-05), CD4^+^ T cells (partial.cor = 0.06, *p* = 2.04e-07), macrophages (partial.cor = 0.119, *p* = 1.14e-02), B cells (partial.cor = 0.126, *p* = 7.37e-03), neutrophils (partial.cor = 0.238, *p* = 2.92e-07), and dendritic cells (partial.cor = 0.17, *p* = 3.20e-04). On the other hand, the MCM4 expression in metastatic cutaneous melanoma was significantly correlated with the levels of CD8^+^ T cells (partial.cor = 0.111, *p* = 4.17e-02), CD4^+^ T cells (partial.cor = 0.039, *p* = 4.66e-01), macrophages (partial.cor = 0.049, *p* = 3.60e-01), B cells (partial.cor = 0.045, *p* = 4.00e-01), neutrophils (partial.cor = 0.15, *p* = 4.86e-03), and dendritic cells (partial.cor = 0.116, *p* = 3.16e-02). Together, these results indicated that MCM4 could play a significant role in the regulation of immune cell infiltration in the melanoma tissues.

### 3.2 MCM4 Expression is Correlated with Immune Marker Expression

Further, the TIMER and Gene Expression Profiling Interactive Analysis (GEPIA) databases were employed to explore the relationship between MCM4 expression and the infiltration levels of immune cells in different melanomas based on a series of specific immune markers. Explicitly, the correlation was assessed between MCM4 expression and the levels of specific cell subtype markers, including monocytes, TAMs, as well as M1 and M2 macrophages. It was observed that MCM4 expression was associated with the specific markers based on monocytes (CD86 and CD115), TAMs (CCL2 and IL10), M1 macrophages (INOS, IRF5, and COX2), as well as M2 macrophages (CD163, VSIG4, and MS4A4A). Among these notified markers, significant correlations were observed between MCM4 expression and markers of monocytes (CD86 and CD115), TAMs (CCL2 and IL10), M1 macrophage (INOS, IRF5, and COX2), and M2 macrophage (CD163, VSIG4, and MS4A4A) in melanomas (Fig. **2**).

### 3.3 MCM4 Induces Macrophage Polarization to M2

To further investigate the role of MCM4 in inducing macrophages towards M2 polarization, the human THP-1, and the mouse RAW264.7 cell lines were employed as normal monocytic macrophage cell lines. On the one hand, THP-1 cells were incubated with phorbol 12-myristate 13-acetate (PMA) for their differentiation into M0 macrophages, characterized by adherent morphology. On the other hand, lipopolysaccharide (LPS) was used to differentiate RAW264.7 cells into M1 macrophages, characterized by adherent morphology and protruding pseudopodia (Fig. **3A**). As anticipated, macrophages transfected with siMCM4 showed a significant increase in the expression of M2 markers (CD86 and iNOS) compared to the control treatment group. Contrarily, the expression level of the M1 marker iNOS was significantly decreased compared to the control treatment group (Fig. **3B**).

### 3.4 Exosomal Extracts are Systematically Characterized

The extracted exosomal vesicles were initially characterized by observing the morphology under TEM and determining the particle size using the nanoparticle size analyzer. It was observed from the TEM observations that exosomes showed typical morphology of spherical or elliptical shapes (Fig. **3C-a**). Similar to TEM observations, the typical diameter of the extracted vesicles by nanoparticle analyzer was indicated in the range of 50 to 110 nm (Fig. **3C-b**), which was within the typical diameter range of exosomes (30-150 nm). Typically, exosomes are known to be enriched with specific proteins, lipids, and RNA in their surface and cargo, which can be commonly used exosomal markers. Accordingly, the Western blot analysis demonstrated the expression levels of various specific proteins, such as TSG101, CD81, CD9, and CD63. These results showed positive expression of CD81 and CD63 proteins, while the expression of Calnexin protein was negative. In contrast, the expression of the same markers in cells showed the opposite pattern, indicating the expression profiles consistent with the exosomal markers (Figs. **3C-c** and **3C-d**).

### 3.5 Exosomes by MCM4-overexpressed A375 Cells Regulate Migration and Invasion Abilities of HEM-L Cells

In addition to the proliferation ability, the influence of exosomes from MCM4-overexpressed A375 cells on the migration and invasion abilities of the HEM-L cells was demonstrated. After 24 h of treatment with exosomes, the migration ability of the HEM-L cells was evaluated using the scratch assay. It was observed from the analysis that the exosomes from the MCM4-overexpressing group exhibited enhanced cell migration ability compared to the NC group (Figs. **4A** and **a**). Similarly, the invasion capability of the HEM-L cells was assessed using the Transwell assay. The exosomes from the MCM4-overexpressing group of A375 cells displayed increased invasion ability compared to the NC treatment group (Figs. **4B** and **b**, *p* < 0.05). These findings demonstrated that the released exosomes by MCM4-overexpressed A375 cells could substantially regulate the migration and invasion abilities of HEM-L cells.

### 3.6 Exosomes from MCM4-overexpressed A375 Cells Affect the Proliferation of HEM-L Cells

Further, the influence of exosomes derived from MCM4-overexpressed A375 cells on the proliferation of HEM-L cells was investigated. After 24 h of treatment with exosomes, the proliferation ability of HEM-L cells was assessed using the CCK-8 assay. The results revealed that the proliferation ability of the exosomes from the MCM4-overexpressing group was significantly higher compared to the NC group (Fig. **4C**). In addition, the clonogenic formation capability of the cells was evaluated using the colony formation assay. It was observed from the experimental results that the exosomes from the MCM4-overexpressing treatment group exhibited increased clonogenic formation ability compared to the NC group (Fig. **4D**, *p* < 0.05). These findings suggested that exosomes from MCM4-overexpressed A375 cells could affect the proliferation and clonogenic abilities of HEM-L cells.

### 3.7 Exosomes by MCM4-overexpressed A375 Cells Promote the Expression of Related Proteins in HEM-L Cells

Considering the proliferation and migration abilities, the expression levels of specific proteins, such as PCNA, C-MYC, and MMP2, were evaluated by Western blot analysis. The results indicated a significant increase in the expression levels of these notified proteins in the OV-MCM4-Exo treatment group compared to the NC-Exo treatment group. These findings suggested that exosomes derived from MCM4-overexpressing A375 cells could significantly promote the proliferation and migration abilities, as well as the expression levels of specific markers of HEM-L cells (Fig. **4E**).

## 4 DISCUSSION

Within the tumor microenvironment (TME), the dynamic interplay between heterogeneous tumor cells and stromal cells, including immune cells, plays a crucial role in the formation, progression, and therapeutic resistance of tumors [29-31]. Typically, tumor cells within the TME exert regulatory control over other stromal cells through a complex network of signaling pathways and molecular mechanisms, thereby enhancing their capabilities for growth, invasion, and metastasis [32-34]. In this context, the phenotype and functional properties of macrophages, which exhibit significant heterogeneity, are profoundly influenced by the surrounding microenvironment [35, 36]. As pivotal components of the TME, tumor-associated macrophages (TAMs) exhibit diverse and often dualistic roles at different stages of tumor development. These roles include promoting tumor growth, facilitating angiogenesis, enabling tumor invasion and metastasis, and mediating resistance to chemotherapy [34, 37-39]. Given the critical involvement of TAMs in tumor progression, understanding the mechanisms by which tumor cells regulate macrophage polarization is of paramount importance [40, 41]. Previous studies have demonstrated that tumor cells can induce macrophage polarization within the TME through the secretion of soluble molecules, such as cytokines, chemokines, and metabolites [36, 42]. In recent years, extracellular vesicles (EVs), which encompass exosomes, microvesicles, and other membrane-bound particles, have emerged as key mediators of intercellular communication. These vesicles carry a diverse cargo of proteins, lipids, and nucleic acids, including microRNAs (miRNAs) and long non-coding RNAs (lncRNAs), which can modulate the function of recipient cells. Tumor-derived extracellular vesicles have been implicated in the recruitment and reprogramming of TAMs, thereby contributing to the establishment of a pro-tumorigenic microenvironment. For instance, the lncRNA PTENP1, which is transmitted via extracellular vesicles, has been shown to inhibit bladder cancer progression by suppressing the expression of PTEN, a well-known tumor suppressor gene [43]. This highlights the potential of extracellular vesicles as both biomarkers and therapeutic targets in cancer. Furthermore, the bidirectional communication between tumor cells and TAMs through extracellular vesicles underscores the complexity of the TME and the need for a comprehensive understanding of these interactions to develop effective anti-cancer strategies. Future research should focus on elucidating the specific molecular mechanisms by which tumor-derived vesicles modulate macrophage function, as well as exploring the therapeutic potential of targeting these pathways to disrupt tumor progression and enhance treatment efficacy.

MCM4 is one of the key proteins involved in initiating DNA replication, maintaining chromosomal stability, and regulating the cell cycle of cells [44-47]. Nevertheless, aberrant upregulation of MCM4 expression is closely associated with poor prognosis toward the development of various malignant tumors [48, 49]. Previous experimental results suggested that MCM4 might promote unrestricted proliferation and DNA replication in melanoma cells, thereby exacerbating tumor progression. Accordingly, high expression of MCM4 is correlated with the invasiveness and malignancy attributes of melanoma, serving as a prognostic biomarker [50]. To this end, macrophages can polarize into pro-inflammatory (M1) and anti-inflammatory (M2) phenotypes [51]. In this vein, M1 macrophages exhibit anti-tumor properties, while M2 macrophages are typically associated with tumor growth and metastasis [11]. Cytokines and various signaling molecules in the microenvironment of melanomas can induce the polarization of surrounding macrophages towards the M2 phenotype, thereby promoting tumor cell growth and metastasis [52-54]. Specifically, M2 macrophages may support the development of melanoma by secreting required growth factors, promoting angiogenesis, and maintaining an anti-inflammatory microenvironment [55, 56]. In melanoma, MCM4 may alter the secretion of cytokines by tumor cells, further impacting the polarization of neighboring macrophages by significantly affecting cell cycle and proliferation. Understanding this interaction can provide new perspectives toward developing novel therapeutic strategies targeting melanoma, such as targeting MCM4 or modulating macrophage polarization states to inhibit tumor development. Considering this aspect, this study further investigated the association between MCM4 and tumor immune cell infiltration using the TIMER database, providing new insights into exploring the functional role of MCM4 in melanoma. Thus, potential mechanistic foundations were established on MCM4, influencing the interactions between immune cells and tumors.

To this end, the extracellular vesicles released by cells play important roles in intercellular communication, specifically in the TME [19, 21, 57-59]. These vesicles carry various biological molecules, such as proteins [60, 61], lipids, RNA, and DNA, and influence the function of other cells or the surrounding normal tissue [61-64]. Specifically in TME, the tumor cells can promote the growth and survival rate of adjacent tumor cells by delivering growth factors and signaling molecules through these released extracellular vesicles [54, 65, 66]. In addition, these vesicles may carry anti-apoptotic molecules that can help tumor cells resist the therapeutic efficiencies of chemotherapy and radiotherapy [41, 67, 68]. Moreover, these extracellular vesicles can modify the behavior of surrounding normal cells, including fibroblasts, endothelial cells, and immune cells [69-71]. For instance, these cells may induce fibroblasts to produce collagen, thereby altering the physical properties of the tumor stroma [72, 73]. Several growth factors and pro-angiogenic molecules in tumor-derived extracellular vesicles can stimulate the formation of new blood vessels, providing essential oxygen and nutrients to the tumor cells. Further, these biological components may directly interact with the cancer cells or modulate the immune system's response to cancer cells [74, 75]. In this study, the experimental results revealed that knocking down MCM4 in macrophages promoted their polarization towards the M2 phenotype, thus exerting an immunosuppressive effect on the TME and promoting tumor growth. In addition, extracellular vesicles secreted by MCM4-overexpressing melanoma A375 cells could enhance the biological behavior of normal melanocytes (HEM-L cells), including increased proliferation, migration, and invasion abilities. Considering these experimental results, we believe that the molecular composition of tumor-derived extracellular vesicles can be used to predict tumor type and stage, making them potential non-invasive biomarkers for tumor diagnosis and monitoring.

## CONCLUSION

This study aims to delve into the role of MCM4 in melanoma progression, with a particular focus on regulatory communication between macrophages and cancer cells mediated by extracellular vesicles (EVs). This study preliminarily revealed the key role of extracellular vesicles derived from melanoma cells in establishing immunosuppressive tumor microenvironment (TME) and promoting tumor progression. These vesicles are small membrane-bound particles released by melanoma cells that carry a variety of bioactive molecules, including proteins, lipids, and nucleic acids, capable of regulating the function of immune cells within TME. Specifically, studies have shown that melanoma-derived EVs may inhibit the activity of cytotoxic T cells, promote differentiation of regulatory T cells (Tregs), and enhance the inhibitory function of myeloid derived suppressor cells (MDSCs). In addition, these EVs may also promote extracellular matrix (ECM) remodeling and angiogenesis, further supporting tumor growth and metastasis. However, as promising as these findings are, they are still in the early stages of research. Numerous experimental and clinical studies are needed to further explore the specific mechanisms of these interactions. Future research should aim to identify key molecular components that drive immunosuppression in melanoma-derived EVs, elucidate the relevant signaling pathways, and evaluate the potential of these EVs as a melanoma treatment strategy. In addition, longitudinal clinical studies are critical to determine the prognostic and predictive value of EVS-based biomarkers in melanoma patients. Only by fully understanding the complex interaction between melanoma-derived EVs and TME will we be able to develop more effective therapeutic interventions to combat this aggressive skin cancer. Only two macrophage cell lines (THP-1 and RAW264.7) were used in this study, which limits the generalisability of the results to other macrophage populations or conditions in vivo. The conclusion that MCM4 promotes macrophage M2 polarization and enhances melanoma cell proliferation, migration, and invasion is based on limited experimental evidence and a lack of in-depth understanding of the mechanism. In order to make up for the limitations of this study, the future research direction should be more comprehensive and detailed. For example, extending studies to a broader range of macrophage cell lines and, more importantly, adopting in vivo models to better understand the role of MCM4 in melanoma progression in a physiologically relevant context, as well as the role of other proteins or signaling pathways that may be involved in the regulation of macrophage polarization and exosome secretion, to more fully analyze extracellular vesicles, To understand their role in communication between macrophages and melanoma cells.

## Figures and Tables

**Fig. (1):**
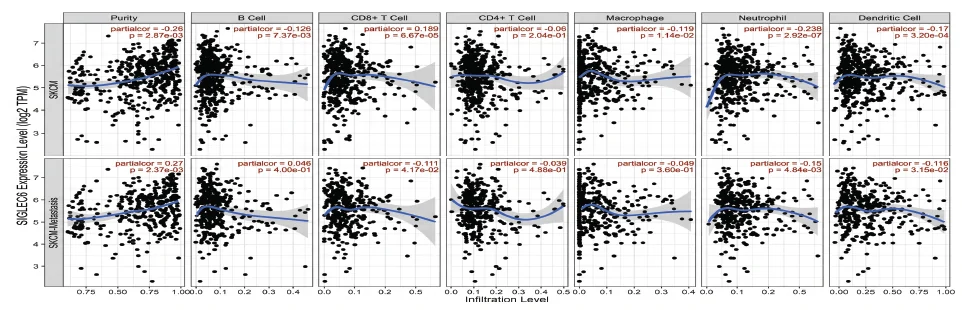
The TIMER database indicates the correlation between MCM4 expression and infiltration levels of various immune cells in cutaneous and metastatic cutaneous melanomas.

**Fig. (2):**
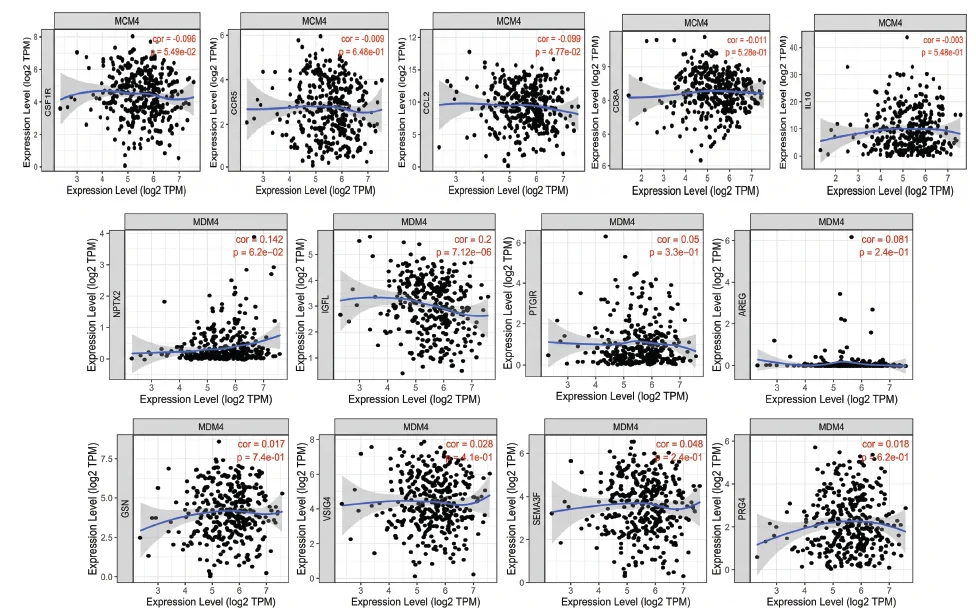
The scatter plots show the correlations between MCM4 expression level in melanomas and markers of monocytes, TAM, as well as M1 and M2 macrophages.

**Fig. (3):**
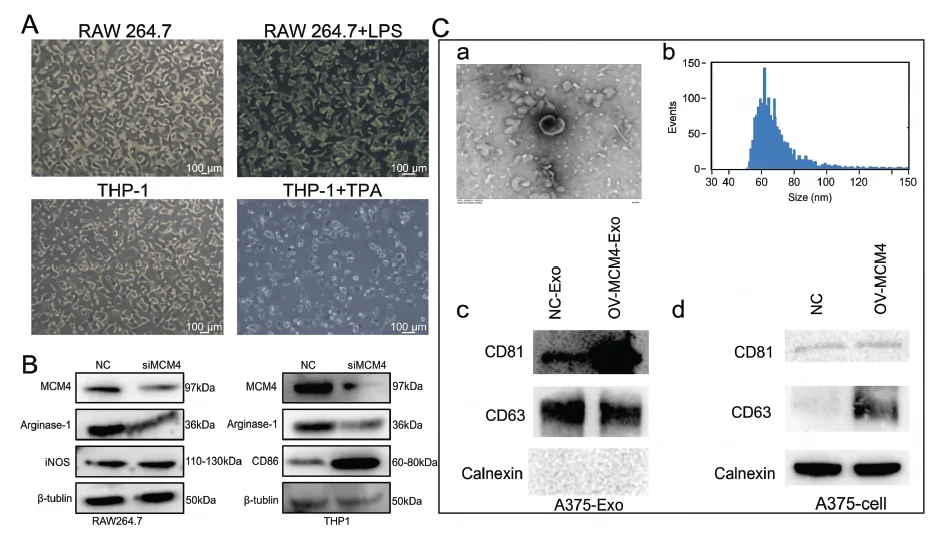
(**A**) The images show the morphology of macrophages before and after induced polarization. (**B**) Western blot analysis indicates the relationship between the changes of MCM4 and the expression levels of M1 and M2 macrophage marker proteins. (C-a) TEM observations show the exosome morphological structure. (C-b) The nanoparticle size analyzer presents the exosome diameter analysis. (C-c, C-d) Western blot analysis describes the expression levels of various specific exosome-related markers.

**Fig. (4):**
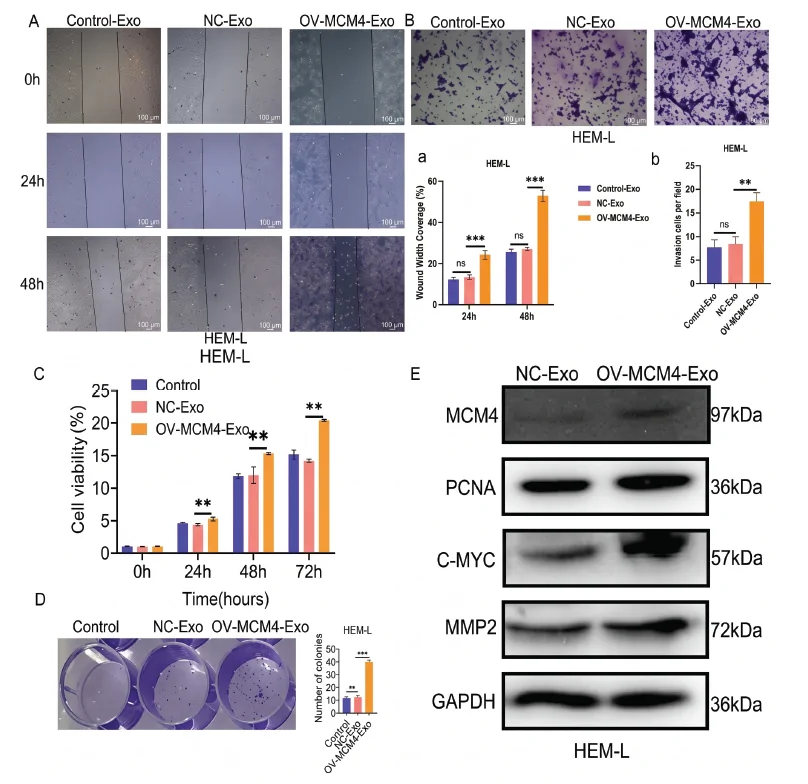
The data present (**A**) migration capability by scratch and (**B**) invasion ability of HEM-L cells by Transwell assay of exosomes produced by MCM4-overexpressed A375 cells. The data indicate the effect of exosomes produced by A375 cells overexpressing MCM4 on the proliferation ability of HEM-L cells by (**C**) CCK-8 and (**D**) plate cloning assays. (**E**) Western blot assay detects changes in the expression levels of various specific proteins of HEM-L cells after the action of exosomes produced by MCM4-overexpressed A375 cells.

## Data Availability

The datasets of single-gene bioinformatics analysis analyzed during the current study are available in the TIMER database (https://cistrome.shinyapps.io/timer/).

## LIST OF ABBREVIATIONS

TME: Tumor Microenvironment
TAMs: Tumor-associated Macrophages
TGF-β: Transforming Growth Factor-beta
OS: Overall Survival
PFS: Progression-free Survival
DNA: Deoxyribonucleic Acid
RNA: Ribonucleic Acid
