# Supplementary material for: Promotion of Melanoma Progression through MCM4-Induced Immune Suppression and Polarization of Macrophages by Carcinogenic Exosomes
Source: Curr Cancer Drug Targets. 2025 May 14;26(8):946–56. doi: 10.2174/0115680096357750250507073948 (PMC13678606; doi:10.2174/0115680096357750250507073948)
Supplement: Supplementary file 1 [file CCDT-26-8-946_SD1.pdf]

Supplementary Material

Promotion of Melanoma Progression through MCM4-Induced Immune Suppression and Polarization of Macrophages by Carcinogenic Exosomes

Xuewei Zhang[1#], Sirong Liu[1], Deni Kang[1] and Ronghua Yang[1,*]

[1]*Department of Burn and Plastic Surgery, Guangzhou First People's Hospital, Guangzhou Medical University, Guangzhou 510000, Guangdong, China*

Table SI. The target sequence of short hairpin RNA MCM4.

Gene name	sense	antisense
MCM4-Mus-1309	GCAGACACCUCACACUAUUTT	AAUAGUGUGAGGUGUCUGCTT
MCM4-Mus-2126	CCGCACACAUUGUUGUCAATT	UUGACAACAAUGUGUGCGGTT
MCM4-Homo-610	GCAGAAGAUAUAGUGGCAATT	UUGCCACUAUAUCUUCUGCTT
MCM4-Homo-1793	GCCAGUACACGUCUGGGAATT	UUCCCAGACGUGUACUGGCTT

Protein Concentration was Detected by BCA Method

0.8ml protein standard solution was added into a tubulin standard (20mg BSA), which was fully dissolved and prepared into 25mg/ml protein standard solution. Take an appropriate amount of 25mg/ml protein and dilute it to 0.5mg/ml. According to the number of samples, 50 volumes of BCA reagent A and 1 volume of BCA reagent B(50:1) were used to prepare an appropriate amount of BCA working liquid, and thoroughly mixed. The standard was added 0, 1, 2, 4, 8, 12, 16, 20μl to the standard well of the 96-well plate, and the standard diluent was added to make up to 20μl, equivalent to the standard concentration of 0, 0.025, 0.05, 0.1, 0.2, 0.3, 0.4, 0.5mg/ml, respectively. Add the appropriate volume of sample to the sample hole of the 96-well plate. Add 200μl BCA working solution to each well and place for 20-30 min at 37℃. The absorbance of A562, or wavelengths between 540-595nm, was measured with an enzyme marker. The protein concentration of the sample was calculated from the standard curve and the sample volume used. Dilute the protein sample with 5×Loading Buffer and PBS according to the concentration, so that every 15μl volume contains 15-30μg protein sample. Boiling water bath for 10 minutes to protein white sample solution.

Table SII. The antibodies involved in the article.

Antibody	Catalog number	Molecular weight	Dilution	Supplier
MCM4	67103-1-Ig	97kDa	1:5000	Proteintech
PCNA	ab92552	36 kDa	1:5000	abcam
MMP2	Ab86607	75kDa	1:1000	abcam
C-MYC	67447-1-Ig	55-60kDa	1:1000	Proteintech
CD81	56039	22kDa	1:1000	CST
CD63	52090	25-60kDa	1:1000	CST
Calnexin	2433	90kDa	1:1000	CST
Arginase-1	16001	36kDa	1:5000	Proteintech
CD86	13395-1	60-80kDa	1:2000	Proteintech
iNOS	22226-1	110-130kDa	1:1000	Proteintech
GAPDH	60004-1-Ig	36kDa	1:50000	Proteintech
β-tublin	10094-1	50kDa	1:5000	Proteintech
